# Neoadjuvant Immunotherapy for Hepatocellular Carcinoma Is Associated with Improved Survival After Hepatectomy: A Matched Analysis of the 2017 to 2022 National Cancer Database †

**DOI:** 10.3390/cancers18132137

**Published:** 2026-07-01

**Authors:** Lawrence Chiang, Adam Bodzin, Daniel Lin, Nader Hanna, Scott Koeneman, Hien Dang, Charles J. Yeo, Christopher Shubert, Richard Zheng

**Affiliations:** 1 Department of Surgery, Thomas Jefferson University Hospital, Philadelphia, PA 19107, USA; lxc386@students.jefferson.edu (L.C.); adam.bodzin@jefferson.edu (A.B.); nader.hanna@jefferson.edu (N.H.); scott.koeneman@jefferson.edu (S.K.); hien.dang@jefferson.edu (H.D.); charles.yeo@jefferson.edu (C.J.Y.); 2 Department of Medicine, Division of Medical Oncology, Thomas Jefferson University Hospital, Philadelphia, PA 19107, USA; daniel.lin@jefferson.edu; 3 Department of Surgery, Division of Surgical Oncology, Johns Hopkins Hospital, Johns Hopkins University, Baltimore, MD 21287, USA

**Keywords:** hepatocellular carcinoma, neoadjuvant immunotherapy, National Cancer Database

## Abstract

**Background:** Effects of neoadjuvant immunotherapy (NIT) before liver resection are not well-described in hepatocellular carcinoma (HCC). Survival and R0 resection rates in HCC patients undergoing curative-intent resection with or without NIT were evaluated. **Methods:** A retrospective study of stage I-III HCC patients undergoing hepatectomy from the 2017–2022 National Cancer Database was performed. Patients receiving NIT were exactly matched 1:3 to non-NIT patients by clinical stage. Primary outcomes were margin positivity, 30- and 90-day mortality, and overall survival. **Results:** After matching 510 patients, 97 received NIT and 291 did not (non-NIT). Resections included segmental (178, 45.9%), lobar (149, 38.4%), and extended hepatectomy (61, 15.7%). The NIT group included ablation (6, 6.2%), radiation (25, 25.8%), and Y90 (18, 18.6%). The non-NIT group included 124 (42.6%) chemotherapy, 23 (7.9%) chemotherapy plus ablation, 31 (10.7%) chemotherapy plus radiation, four (1.4%) radiation, and 109 (37.5%) upfront surgery. NIT patients were treated more at high-volume (41.2% vs. 0%) and academic/research centers (85.4% vs. 50.5%). NIT R0 resection rates were higher (97.9% vs. 93.2%, *p* = 0.009). Median survival was higher in the NIT group (59.1 vs. 50.4 months, *p* = 0.002); 30- and 90-day mortality were similar. NIT was not independently associated with improved survival in multivariable Cox regression (HR 0.73, 95% CI [0.35, 1.55]), although the effect estimate indicates a protective effect may be present. **Conclusions:** NIT was associated with higher R0 resection rates and overall survival without increasing short-term mortality in selected patients at high-volume academic centers, but was not independently associated with survival when adjusting for confounders. As we continue to see increasing use of immunotherapy in the neoadjuvant setting, these findings should be considered hypothesis-generating and warrant prospective validation before widespread adoption for HCC.

## Simple Summary

The role of neoadjuvant immunotherapy (NIT) for hepatocellular carcinoma (HCC) is not well described. A matched retrospective study using the 2017–2022 National Cancer Database was performed to compare outcomes of stage I-III HCC NIT and non-NIT patients undergoing hepatectomy. Patients who received NIT had higher rates of margin-negative resection and longer overall survival without an increase in short-term postoperative mortality, although these benefits were not statistically significant after adjusting for potential confounders. NIT was more commonly utilized in high-volume academic centers. These findings should be considered hypothesis generating with further prospective studies needed to verify whether NIT may have a clinical benefit in HCC patients.

## 1. Introduction

Hepatocellular carcinoma (HCC) is the predominant form of liver cancer globally, the sixth most frequently diagnosed cancer worldwide, and the sixth leading cause of cancer-related death in the United States as of 2023 [1]. The worldwide incidence of HCC has increased 70% between 1990 and 2019, and in the United States, the age-standardized HCC-specific mortality rate increased from 3.7 to 5.0 per 100,000 persons between 2006 and 2022 [2,3]. The incidence and mortality rates of HCC have increased significantly over the past decades, primarily due to increased rates of alcohol-associated liver disease (seen in 37% of HCC patients) and metabolic dysfunction-associated liver disease (seen in 23% of HCC patients) [4]. Due to non-optimal screening strategies, over 60% of HCC patients present with advanced-stage disease and are surgically unresectable, either due to distant metastases and/or locoregional involvement [5]. For those with locally advanced disease, neoadjuvant systemic chemotherapy and/or locoregional therapies have been used to attempt to clinically downstage unresectable tumors. However, among the multitude of treatment options available for advanced HCC, there is little evidence to suggest that one treatment sequence is more effective than the other in prolonging survival or downstaging tumors prior to surgery.

For years, effective systemic therapy consisted primarily of tyrosine kinase inhibitors (TKIs) such as sorafenib, as outcomes with traditional cytotoxic chemotherapy were dismal [6]. More recently, immune checkpoint inhibitors such as atezolizumab and durvalumab have been approved for use in unresectable HCC [7,8]. The IMbrave050 and 150 trials have expanded the toolkit of HCC treatments by introducing a novel combination immunotherapy regimen in the unresectable and adjuvant setting, respectively. IMbrave150 demonstrated that atezolizumab plus the VEGF targeting agent bevacizumab significantly improved overall survival and progression-free survival compared to sorafenib in patients with unresectable HCC [7], whereas IMbrave050 initially showed that the same regimen improved recurrence-free survival when used as adjuvant therapy following curative-intent resection or ablation as compared to active surveillance, although this was ultimately a negative trial with longer follow-up [9]. Despite these studies, however, there are still limited data regarding the role of administering immunotherapy for HCC in the neoadjuvant setting prior to resection.

Given the lack of consensus regarding neoadjuvant treatment paradigms for HCC, we used the 2017–2022 National Cancer Database (NCDB)—a nationwide oncology outcomes database consisting of data from over 1500 cancer programs—to compare survival and margin-negative (R0) resection rates between those with HCC undergoing partial hepatectomy receiving neoadjuvant immunotherapy (NIT) and those who did not receive neoadjuvant immunotherapy (non-NIT). The NCDB provides a large sample of patients with HCC who have received immunotherapy that is not available through any single-institution experience, reflecting the introduction of potentially efficacious immunotherapy regimens for HCC. We hypothesized that NIT is associated with both improved overall survival and increased margin-negative resection rates when compared to historical controls.

This study extends a conference presentation previously presented at the 2025 American College of Surgeons Clinical Congress [10].

## 2. Methods

### 2.1. Data Source

The NCDB is a clinical oncology database sourced from hospital registry data collected in more than 1500 Commission on Cancer-accredited facilities, capturing roughly 70% of new cancer cases each year in the United States [11]. Data are portioned into organ-specific participant user files (PUFs) containing information on patient- and hospital-level variables and outcomes such as demographics, comorbidity (as represented by Charlson–Deyo score), staging, cancer-related procedures, adjuvant and neoadjuvant therapy, readmissions, 30- and 90-day mortality, hospital type, hospital location, and teaching status. Staging in the NCDB is assigned based upon the *American Joint Commission on Cancer (AJCC) Cancer Staging Manual* edition in use at the time when the case was diagnosed and therefore ranges between the 6th and 8th editions for the years included in the study. Data for this study were extracted from the 2017–2022 *Liver and Intrahepatic Bile Ducts* NCDB participant user file (PUF).

### 2.2. Study Population

**Figure 1:**
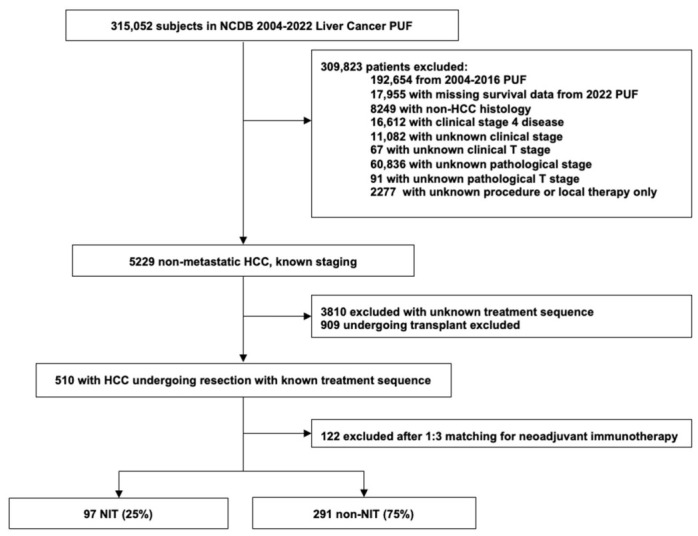
Flow diagram of study population. HCC: hepatocellular carcinoma; NCDB: National Cancer Database; NIT: neoadjuvant immunotherapy; PUF: participant user file. Staging was assigned according to the American Joint Committee on Cancer 6th–8th editions for the year in which NCDB data were recorded.

Subjects were eligible to be included in the study if they were diagnosed with clinical stage I-III HCC according to the AJCC staging system and underwent resection with curative intent [12]. Clinical staging was assigned at initial diagnosis, prior to treatment. Subjects undergoing liver transplant were excluded. Patients who only underwent ablation or other locoregional therapies alone (i.e., chemoembolization or radioembolization) were excluded, but patients who had multiple modalities of treatment were included if curative intent resection was also performed. Subjects were excluded if their pathological TNM stage or components thereof were unknown or missing. Subjects with unknown treatment sequences were also excluded. Remaining subjects were then stratified by receipt of neoadjuvant therapy, which was determined via a single variable (RX_SUMM_SYSTEMIC_SUR_SEQ) which encodes the sequence of systemic therapy relative to surgery. A flow diagram of inclusion/exclusion criteria and the final study cohort is displayed in Figure 1.

### 2.3. Defining Non-Surgical Therapies

Non-surgical treatments performed for HCC include systemic chemotherapy, immunotherapy, transarterial chemoembolization (TACE), transarterial radioembolization (TARE) [13], hepatic lesion ablation, and external beam radiation. The specific agents used for chemotherapy or TACE are not defined within the NCDB. Similarly, the NCDB does not differentiate between chemotherapy given systemically or as TACE, although given trends in treatment patterns across the United States at the time of this study and the proven effectiveness of TACE for patients with initially unresectable HCC, we assume that most of the chemotherapy given in the neoadjuvant setting is likely administered as TACE [14,15]. Treatment codes for radiation brachytherapy were used to identify patients undergoing TARE. Ablation is recorded as a separate surgical procedure. However, as the NCDB only records the date of the most definitive cancer procedure (i.e., surgery), patients receiving multiple surgical procedures prior to definitive resection were considered to have likely been ablated before surgery. Immunotherapy is categorized as systemic therapy but is coded separately from traditional cytotoxic chemotherapy.

### 2.4. Outcomes

Primary outcomes were surgical resection margin positivity, 30- and 90-day mortality, and overall survival. Kaplan–Meier survival curves were generated from these data [16].

### 2.5. Matching

Exact matching was performed to account for stage as a confounder [17]. Exact stage-for-stage matching between the NIT and non-NIT groups was performed on a 1:3 basis, ensuring that there were equal numbers of patients with each clinical stage in each cohort.

### 2.6. Statistical Analyses

Demographic and clinical characteristic data were summarized using standard descriptive statistics. The primary outcomes of margin positivity, 30-day mortality, and 90-day mortality were evaluated using Fisher’s exact test to assess the hypothesis that these outcomes were independent of whether NIT was received or not [18]. Overall survival was evaluated using the log-rank test to assess whether survival over time was equivalent between the two cohorts [19]. An exploratory Cox proportional hazards model to assess the effect of neoadjuvant immunotherapy on postsurgical survival while adjusting for additional covariates was performed while adjusting for the following variables: facility type, sex, age (non-linear spline term), race, insurance, Charlson score (treated as categorical variable), type of surgery, hospital volume by tertile category and days from diagnosis to surgery (non-linear spline term).

As we tested multiple hypotheses, we performed a Bonferroni correction to ensure a family-wise error rate of 0.05 [20]. Thus, each of the four tests detailed above was performed at 0.0125 in order to assess the given hypothesis.

Statistical analysis was performed in Stata v. 19.0 (©StataCorp LLC, College Station, TX, USA) and R version 4.3.1.

## 3. Results

### 3.1. Demographics

There were 315,052 patients in the NCDB 2017–2022 Liver Cancer PUF. After applying our exclusion criteria, there were 510 eligible patients. After exact 3:1 stage-for-stage matching, there were 388 patients included in the study, with 97 patients in the NIT group and 291 in the non-NIT group (Figure 1). Median age at diagnosis was 65 and 66 years old in the NIT and non-NIT groups, respectively. Subjects were predominantly male, making up 79.4% and 71.1% of the NIT and non-NIT groups, respectively. The majority of patients were white (54.6% NIT vs. 65% non-NIT). The type of facility in which these patients were treated varied by treatment group, with 85.4% of the NIT patients being treated at academic centers compared to only 50.5% in the non-NIT group. In addition, when individual centers were categorized into quartiles by their volume of HCC cases per year, none of the non-NIT patients were treated in hospitals in the highest quartile of hospital volume as compared to 41.2% in the NIT group. There were 48 (49.5%) patients who underwent non-anatomic resection and 49 (50.5%) patients who underwent formal anatomic resection in the NIT group. There were 130 (44.7%) patients who underwent non-anatomic resection and 161 (55.3%) patients who underwent formal anatomic resection in the non-NIT group. Based on the median MELD and AFP levels pre-resection, the immunotherapy group seems to have slightly more advanced disease based upon median AFP (93 vs. 25.3) and slightly better liver function based upon median MELD (7.5 vs. 8.17). MELD scores were missing in 26 (26.8%) patients in the NIT group and missing in 83 (28.5%) patients in the non-NIT group. AFP values were missing in 12 (12.3%) patients in the NIT group and missing in 46 (15.8%) patients in the non-NIT group. The demographics of both groups are summarized in Table 1.

### 3.2. Pathological and Survival Outcomes

Pathological and survival outcomes are shown in Table 2. Due to exact stage-for-stage matching being performed, the clinical stages between the groups were identical, with 49.5% stage I, 19.6% stage II, and 30.9% stage III. Margin-negative resection (R0) was seen at significantly higher rates in the NIT group (97.9% vs. 93.2%, *p* = 0.009). Eighty-eight of the NIT patients (90.7%) had incomplete pathological staging and 153 (52.6%) non-NIT patients had incomplete pathological staging. The majority of patients in both the NIT and non-NIT group did not have any lymph nodes examined (80.4% vs. 66.3%).

**Figure 2:**
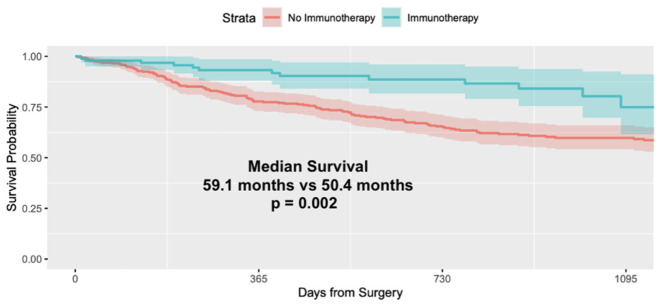
Kaplan Meier 3-Year Overall Survival Curve, by Receipt of Neoadjuvant Immunotherapy. Kaplan–Meier curve demonstrating three-year overall survival after primary resection of hepatocellular carcinoma, stratified by receipt of neoadjuvant immunotherapy.

**Figure 3:**
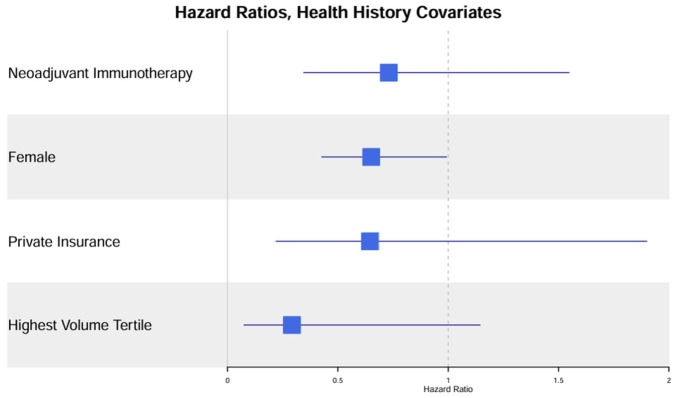
Cox regression model.

NIT patients were associated with higher median survival with an average of 59.1 months compared to 50.4 months in the non-NIT group (log-rank *p* = 002; Figure 2). The 30-day (2.1% NIT vs. 2.4% non-NIT, *p* = 1.0) and 90-day (2.1% NIT vs. 5.2% non-NIT, *p* = 0.26) mortality rates were not different between the two groups. In the NIT group, patients were treated with neoadjuvant immunotherapy for an average of 104 days prior to surgery. In the non-NIT group, the mean time from surgery to initiation of adjuvant systemic therapy was 75 days. In our multivariable Cox regression accounting for facility type, sex, age, race, insurance, Charlson score, type of surgery, hospital volume, and time to surgery, NIT was associated with statistically insignificant lower risk of death (HR 0.73, 95% CI [0.35, 1.55]) for NIT (Figure 3).

## 4. Discussion

In this study of the NCDB, we find that NIT before resection of HCC is associated with significantly higher R0 resection rates and longer overall survival without an accompanying increase in short-term mortality when compared to those who did not receive neoadjuvant immunotherapy. Our study additionally finds that neoadjuvant immunotherapy is preferentially utilized in high-volume academic centers. When accounting for variables such as hospital volume, the use of NIT was not significantly associated with improved survival, suggesting that any potential survival benefit of NIT may be difficult to separate out from the known benefit of being treated at high-volume institutions. The use of immunotherapy in the neoadjuvant setting has not yet been incorporated into guidelines of care for HCC, and our results reflect that it is still only used in a small minority of cases. These findings from a retrospective, observational dataset suggest some potential benefit of the use of neoadjuvant immunotherapy for treating HCC in high-volume centers, but the true benefit of this practice still requires extensive validation in prospective studies.

Our findings observe a trend consistent with several other studies which found similar survival benefits of neoadjuvant immunotherapy in HCC patients. In patients with unresectable HCC, the IMBrave150 trial demonstrated improved overall survival with atezolizumab plus bevacizumab compared to sorafenib. Studies in unresectable HCC after this landmark trial further demonstrated the benefits of NIT in this patient population. Nakazawa et al. conducted a retrospective analysis and found that high-risk advanced HCC patients who received neoadjuvant immunotherapy, many of whom were initially unresectable, achieved similar rates of recurrence-free survival and margin-negative resection as those who underwent upfront surgical resection [21]. In addition, there has been a survival benefit with upfront immunotherapy regardless of downstaging. A phase III clinical trial by Abou-Alfa et al. demonstrated that the combination therapy of tremelimumab plus durvalumab in unresectable HCC patients was superior to sorafenib in prolonging overall survival [8]. A separate randomized controlled trial by Yau et al. also found that adding ipilimumab to nivolumab improved clinical outcomes in patients with advanced hepatocellular carcinoma previously treated with sorafenib [22].

Within the context of resectable HCC, a phase II clinical trial by Marron et al. found that the use of a PD-1 inhibitor (cemiplimab) in the neoadjuvant setting was associated with lower postoperative recurrence rates [23]. These clinical trials demonstrate promise for the use of immunotherapy in both unresectable and resectable HCC. Our study is consistent with the current literature, finding an association between immunotherapy, margin-negative resection, and survival in the neoadjuvant setting. Although neoadjuvant immunotherapy has not yet been incorporated into the standard-of-care for resectable HCC, prior studies have established a precedent for its use. We see a similar trend but are not able to infer causality in this retrospective study.

Our study also highlights the differences in HCC treatment between different institutions, specifically showing that NIT was utilized more often in high-volume academic centers which may be earlier to adopt new paradigms of treatment [24]. Differences in institutional experience and increased multidisciplinary care may be a significant driver for improved outcomes. Several other studies have also shown results consistent with this across multiple different cancers. Elbahrawy et al. found that neoadjuvant chemoimmunotherapy was utilized less frequently in community centers compared to academic centers in patients with non-small-cell lung cancer [25]. In another study by Freeman et al., early-stage triple negative breast cancer patients treated in academic/research programs were more likely to receive neoadjuvant immunotherapy than community programs [26]. These trends may be reflective of differences in the practice of academic centers, which may be presumably higher-volume with more specific expertise than non-academic centers. Although the use of immunotherapy has shown promise in the field of oncology, it is still only used in the minority of hepatocellular carcinoma cases overall.

The idea of using neoadjuvant immunotherapy in HCC is attractive and gaining traction worldwide for several reasons. It is thought that the benefits of giving neoadjuvant immunotherapy are twofold: it may downstage unresectable or locally advanced HCC to be within transplant or resection criteria, or it may select for a better disease biology with a higher tumor mutational burden that may also be responsive to other treatments. As such, the efficacy of neoadjuvant immunotherapy for HCC is also currently being explored in many other clinical trials which remain active at the time of writing this paper. The ImmunoXXL study (NCT05879328) is designed to evaluate liver transplantation outcomes in HCC patients after partial or complete downstaging with atezolizumab and bevacizumab. The YANG-LIFT-HCC trial (NCT07489976) and NCT07059494 both seek to assess response after the combination of neoadjuvant immunotherapy with locoregional liver-directed therapies such as Y90. NCT04974281 aims to assess the efficacy of combining a PD-1 inhibitor with lenvatinib and TACE. Results from these ongoing studies could potentially validate the signal for neoadjuvant immunotherapy that we have observed and may also direct how neoadjuvant immunotherapy could be most effectively used.

There are several limitations in this study, several of which are inherent in utilizing the NCDB. First, these retrospective data capture about 70% of cancer cases in the U.S. and therefore is not fully nationally representative [27,28]. Second, the NCBD lacks granularity with a high prevalence of missing clinical data points (e.g., major venous involvement, unilobar vs. bilobar disease, specific agents used for chemotherapy or TACE) and lacks data on recurrence [29].

In particular, the NCDB had incomplete pathological staging information in 90.7% of NIT patients and 52.6% of non-NIT patients. As a result, this degree of missing data limits our ability to evaluate pathological response, such as downstaging and nodal involvement. Thus, while observed differences in R0 resection rates are significant in our study, conclusions regarding pathological treatment effects should be interpreted conservatively. Additionally, there is no information on the exact regimen or intent/indications for neoadjuvant therapy, and we cannot separate systemic therapy from TACE using the NCDB. Many clinicians treating HCC may base their clinical decision-making upon a different clinical staging system than the AJCC TNM staging which is not easily extrapolated from NCDB data. Immortal time bias also heavily favors the neoadjuvant group and there is more diversity in the treatments used in the non-NIT group. Lastly, it is hard to separate out whether or not immunotherapy is mainly responsible for the impressive effect on survival or if it is a proxy for receiving treatment at high-volume institutions that are experienced in providing multidisciplinary care for HCC. This will require further study as the use of immunotherapy in HCC treatment becomes more commonplace and less limited to selected centers that may be early adopters. As our study is a retrospective observational study using a large database lacking granularity, additional confounding factors may persist despite matching. Therefore, the associations found in this study should not be interpreted as causal evidence that NIT directly improves survival or resection outcomes sufficient to influence practice but rather as supportive preliminary evidence for future prospective trials.

## 5. Conclusions

Our study demonstrates the possibility that using neoadjuvant immunotherapy in stage I to III hepatocellular carcinoma patients may be associated with higher margin-negative resection rates and longer overall survival without affecting short-term mortality, though these effects may be confounded by hospital volume and patient-related factors. Although immunotherapy has been incorporated into treatment paradigms for locally advanced and unresectable HCC, we should take pause before widespread adoption of this practice for resectable HCC. A randomized, prospective study examining survival and downstaging with neoadjuvant immunotherapy in patients with non-metastatic HCC outside of traditional resection or transplant criteria may better help inform changes in clinical practice.

## Figures and Tables

**Table 1:** Demographics after resection of HCC, by neoadjuvant immunotherapy.

	NIT	(%)	Non-NIT	(%)	*p*-Value
	(n = 97)		(n = 291)		
Median Age at Diagnosis (years)	65.0	-	66.0	-	-
Male	77	79.4	207	71.1	-
Race					-
White	53	54.6	189	65.0	
Black	19	19.6	47	16.2	
Asian	23	23.7	45	15.5	
Unknown/Other	2	2.1	10		
Insurance Status					-
Not Insured	3	3.1	7	2.4	
Private Insurance/Managed Care	30	31.3	105	36.3	
Medicaid	15	15.6	30	10.4	
Medicare	46	47.9	142	49.1	
Other	2	2.1	5	1.7	
Facility Type					-
Community Center	0	0.0	7	2.5	
Comprehensive Community	7	7.3	67	23.7	
Academic/Research	82	85.4	143	50.5	
Integrated Network Cancer	7	7.3	66	23.3	
Hospital Volume					-
Lowest Quartile (<25th %ile)	41	42.3	291	100.0	
Middle Quartiles (25–75th %ile)	16	16.5	0	0.0	
Highest Quartile (>75th %ile)	40	41.2	0	0.0	
Charlson–Deyo Score					-
0	46	47.5	157	54.0	
1	33	34.0	56	19.2	
2	11	11.3	44	15.1	
3 or Greater	7	7.2	34	11.7	
T stage					-
T1	48	49.5	144	49.5	
T2	19	19.6	56	19.3	
T3	18	18.6	67	23.0	
T4	12	12.4	24	8.2	
AJCC Clinical Stage Group					-
I	48	49.5	144	49.5	
II	19	19.6	57	19.6	
III	30	30.9	90	30.9	
Neoadjuvant Chemotherapy Alone	0	0.0	124	42.6	-
Neoadjuvant Chemotherapy and Ablation	0	0.0	23	7.9	
Neoadjuvant Chemotherapy and Radiation	0	0.0	31	10.7	
Neoadjuvant Y90 Alone	0	0.0	4	1.4	
Upfront Surgery	0	0.0	109	37,5	
Neoadjuvant Immunotherapy and Ablation	6	6.2	0	0.0	
Neoadjuvant Immunotherapy and Radiation	25	25.8	0	0.0	
Neoadjuvant Immunotherapy and Y90	18	18.6	0	0.0	
Adjuvant Systemic Therapy	32	33.0	117	40.2	-
Adjuvant Immunotherapy	0	0.0	45	15.5	-
Median MELD Score	7.5	-	8.17	-	-
Median AFP Levels	93	-	25.3	-	-
Surgical Procedure					-
Wedge	47	48.5	119	40.9	
Wedge and Ablation	1	1.0	11	3.8	
Lobectomy	38	39.2	104	35.7	
Lobectomy and Ablation	2	2.1	5	1.7	
Extended Hepatectomy	8	8.2	50	17.2	
Extended Hepatectomy and Ablation	1	1.0	2	0.7	

AFP: Alpha-fetoprotein; AJCC: American Joint Commission on Cancer; HCC: hepatocellular carcinoma; MELD: model for end-stage liver disease; NIT: neoadjuvant immunotherapy.

**Table 2:** Outcomes after resection of HCC, by neoadjuvant immunotherapy.

	NIT	(%)	No NIT	(%)	*p*-Value
	(n = 97)		(n = 291)		
Mean days from diagnosis to surgery	169.81 +/− 12.6	-	128.75 +/− 7.7	-	<0.001
Nodal involvement					0.03
No nodal involvement	18	18.6	88	30.3	
Nodal involvement	1	1.0	9	3.1	
No nodes examined	78	80.4	193	66.6	
Tumor grade					0.810
Well differentiated	10	10.3	34	11.7	
Moderately differentiated	45	46.4	155	53.3	
Poorly differentiated	22	22.7	63	21.6	
Undifferentiated	1	1.0	2	0.7	
Not determined or stated	19	19.6	37	12.7	
Margin status					0.009
R0	92	97.9	248	93.2	
R1, R2	2	2.1	18	6.8	
30-day mortality	2	2.1	7	2.4	1.000
90-day mortality	2	2.1	15	5.2	0.261
Post-diagnosis median survival (months)	59.1	-	50.4	-	0.002

NIT: neoadjuvant immunotherapy.

## Data Availability

The data presented in this study are available on request from the corresponding author.
